# COVID-19 induced changes in physical activity patterns, screen time and sleep among Swedish adolescents - a cohort study

**DOI:** 10.1186/s12889-023-15282-x

**Published:** 2023-02-23

**Authors:** Björg Helgadóttir, Andreas Fröberg, Karin Kjellenberg, Örjan Ekblom, Gisela Nyberg

**Affiliations:** 1 The Swedish School of Sport and Health Sciences, Lidingövägen 1, SE-114 33 Stockholm, Sweden; 2 Department of Clinical Neuroscience, Division of Insurance Medicine, Karolinska Institutet, SE-171 77 Stockholm, Sweden; 3 Department of Food and Nutrition, and Sport Science, University of Gothenburg, Läroverksgatan 5, SE-411 22 Gothenburg, Sweden; 4 Department of Global Public Health, Karolinska Institutet, SE-171 77 Stockholm, Sweden

**Keywords:** Sedentary behaviour, Exercise, Accelerometer, Gender, Pandemic, Socioeconomic status

## Abstract

**Background:**

The coronavirus disease 2019 (COVID-19) pandemic had a huge impact on daily life, even in countries such as Sweden where the restrictions were relatively mild. This paper assesses the effects of the COVID-19 pandemic restrictions on physical activity (PA) patterns, screen time, and sleep among Swedish adolescents. The exposures explored include gender, parental education, anthropometrics, and cardiovascular fitness (CVF).

**Methods:**

Cohort data were collected from September 26th to December 6th, 2019, and from April 12th to June 9th, 2021. Participants were 13–14 years-old (7th graders) at baseline with 585 participating at both baseline and follow-up. At both baseline and follow-up PA and sedentary time were measured with accelerometers, and sleep and screen time with questionnaires. The exposure variables (gender, parental education, anthropometrics and CVF) were collected at baseline. Multilevel linear regression analyses were performed.

**Results:**

Moderate-to-vigorous-physical activity (MVPA) remained unchanged while light physical activity (LiPA) decreased and sedentary time increased. Sleep duration decreased and screen time increased. Girls, adolescents with overweight/obesity (BMI and percent body fat), and those with lower CVF at baseline had less favourable changes in PA patterns, sleep and screen time.

**Conclusions:**

Although no significant (α = 0.05) changes were seen in MVPA, both LiPA and sedentary time as well as sleep and screen time changed in unfavourable ways. More intense activities are often organised and seem to have withstood the pandemic, while less intense activities decreased. Some groups were more vulnerable and will need directed intervention in the post-pandemic period as well as when future pandemics hit.

**Supplementary Information:**

The online version contains supplementary material available at 10.1186/s12889-023-15282-x.

## Introduction

The health benefits of physical activity (PA) and sleep, and the detrimental effects of sedentary behaviour, especially screen time, among adolescents have been documented [1–3]. Many national and international PA guidelines, as well as sedentary behaviour and sleep are available [4]. The WHO recommends ≥60 min of Moderate-to-vigorous-physical activity (MVPA) per day for adolescents [5]. Furthermore, the 24 h movement guidelines also recommend limiting prolonged sitting, engage in < 2 h per day of recreational screen time, and sleep for 9–11 h (5–13 y) and 8–10 h (14–17 y) per night [6].

In March 2020, the coronavirus disease (COVID-19) was declared a pandemic. Ever since its outbreak, COVID-19 has remained a public health concern, and people have been recommended to maintain physical distancing, avoid crowded places, and stay home when experiencing illness or symptoms thereof. Some countries and regions have also implemented closures of schools and workplaces, restrictions on mass gatherings, and lockdown [7].

Although critical for preventing the spread of the coronavirus, there has been concern that restriction policies would have negative implications on other health-related factors, such as PA patterns, screen time and sleep among adolescents. Despite recommendations on how to engage in safe PA during the pandemic [8, 9], studies from several countries and regions have reported decreases in PA among children and adolescents [10, 11]. However, virtually all studies to date rely on self-reported PA data [10, 11]. Studies have reported an increase in academic and non-academic screen time [10, 12]. and a change in sleep habits with a shift to later bed- and waking time as well as both positive and negative changes in sleep duration and quality [10].

In contrast to many other countries and regions, Sweden kept society open during the pandemic. Rather than implementing lockdown, the recommendations have been to keep physical distance, avoid large gatherings and non-essential travel, and stay at home and avoid social contacts when ill [13]. Regulations applied to indoor premises, such as sports facilities have led to cancelled leisure-time activities, such as organised sport [13]. Public transportation remained accessible and basic services, such as grocery stores, and primary care a were open. Teaching has partly been conducted remotely in Swedish primary and secondary schools [14].

To date, little is known regarding COVID-19-induced changes in PA patterns, screen time, and sleep among children and adolescents in Sweden. One cross-sectional study used data from questionnaires and found that 20% of the approximately 8300 participating children and adolescents (4–17 y) reported that their PA had decreased [15]. Another Swedish study showed no differences in longitudinal changes in PA patterns and sleep between cohorts of adolescents assessed exclusively before the pandemic and a cohort assessed before and during the pandemic (February and November 2020).

To the best of our knowledge, no study has used objective measurements of PA to investigate the effect of Sweden’s relatively liberal pandemic restrictions on PA patterns among adolescents. The first aim of this study was to investigate changes in PA patterns (MVPA, light physical activity (LiPA), sedentary time during weekends, weekdays, school time and leisure time on weekdays), screen time (during weekdays and weekends, and sleep duration (during weekdays and weekends among Swedish adolescents during the COVID-19 pandemic). A second aim was to investigate whether background characteristics, anthropometrics and cardiovascular fitness predicted changes in these variables.

## Methods

**Fig. 1:**
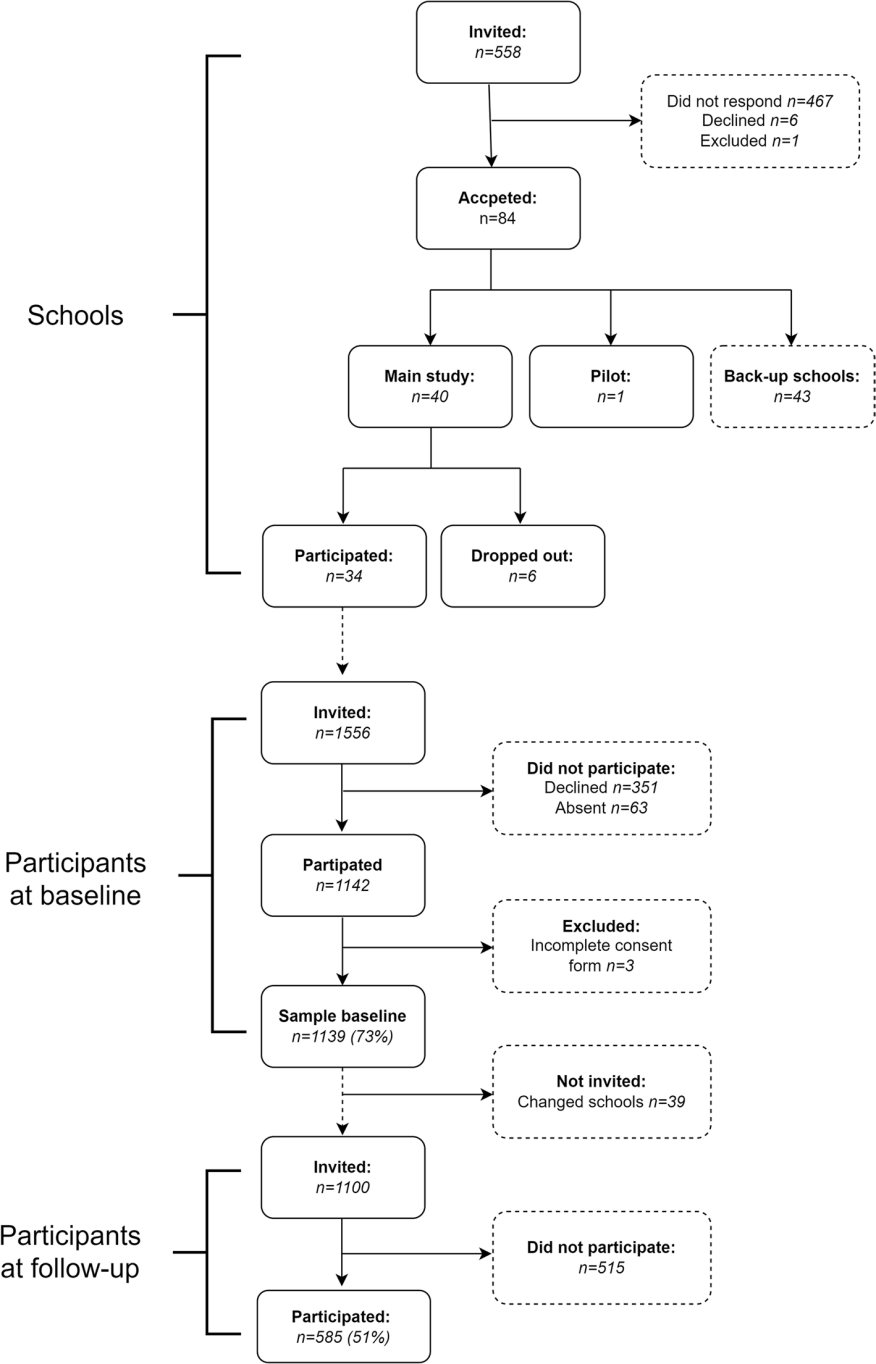
Flow chart of the data collection

Data for this study comes from the project “Physical activity for healthy brain functions in school youth”, a cohort study with measures from September 26th to December 6th 2019 (baseline) and follow-up from April 12th to June 9th 2021. The study was performed in Stockholm, which is the capital of Sweden. The school system in Sweden is deregulated and allows the parents to freely choose between independent and public schools for their children, without paying any fees. The aim for the baseline study was to include at least 1000 participants to be able to explore associations between mental health and physical activity. A convenience sample was used. Schools within a 2–3 hour driving distance from Stockholm, Sweden (ranging from 2 to 200 km away) were invited to participate (*n* = 558). Recruitment was performed from March to June of 2019. Schools with a sports profile, with less than 15 students in grade 7 (13–14-year-olds) and schools with a non-Swedish speaking student population were excluded. Of the 84 schools that showed interest in participating in the study, 40 were chosen to maximize variation in the type of municipality and socioeconomic background of the schools. Six schools dropped out before participating, leaving a total of 34 schools with 1–4 classes of 7th graders participating in each school. Of these 11 (32%) were independent schools and 23 (68%) were public schools and a total of 22 municipalities are represented in the sample, with 44% being located in large cities and municipalities near large cities, 47% medium-sized towns and municipalities near medium-sized towns and 9% in included smaller towns/urban areas and rural municipalities. All students from participating classes were invited to participate (*n* = 1556) and a total of 1139 participated (participation rate 73%). Teachers and principals at each school handled the recruitment of the students in the fall of 2019 and gathered consent forms. The schools were contacted in March 2020 and asked to assist in distributing the questionnaires and accelerometers; 6 schools did not have the time or resources, so questionnaires and accelerometers were sent to the students in these schools directly by e-mail and post. A total of 585 participated at follow-up i.e. either answered the questionnaire or had at least one valid day of accelerometer measurements (51% participation rate), see Fig. 1. The study was pre-registered (ISRCTN15689873) on 17/06/2021.

### Data collection

At baseline, the participants came in groups of at most 60 students per day to the Swedish School of Sport and Health Sciences. The participants arrived in the morning, were informed about the data collection procedures. They answered an electronic questionnaire, had their body composition, height and weight measured, performed a sub-maximal fitness test and were given an accelerometer for recording physical activity to be worn for 1 week. The data collection finished at noon. School schedules of all participating classes were collected.

At follow-up, questionnaires were sent electronically to the participants. In case of non-response after electronic reminders, paper questionnaires were sent out to the home address. Accelerometers were sent out to the schools that agreed to assist with the data collection and given to those willing to participate again. For non-participating schools, the accelerometers were sent directly to the participants after they had answered the electronic questionnaire. The accelerometers were again worn for 1 week. New school schedules for the spring of 2021 were collected. On average 18.4 months (sd 1 month) passed between baseline and follow-up.

### Exposure variables

#### Background variables

In the baseline questionnaire, participants indicated their gender and their parents’ country of birth, classified as “both born in Sweden”, “one born outside and one in Sweden” and “both born outside Sweden”. Register data on parental education for each participant was collected from Statistics Sweden and classified as “12 years or less” and “more than 12 years”, chosen as primary and secondary school combined lasts 12 years in Sweden.

#### Anthropometric measures

Height and weight were measured at baseline using a stadiometer (SECA 5123, SECA Weighing and Measuring Systems) to the nearest mm and a calibrated scale (Tanita BC-418, Tanita Corporation, Tokyo, Japan) to the nearest 0.1 kg. Body mass index (BMI) was calculated (kg/m^2^) and categorised according to sex- and age-specific values from the International Obesity Task Force (IOTF) 2012 [16]. BMI was dichotomised as normal/underweight and overweight/obese. Furthermore, BMI standard deviation score (sds) was calculated, which adjusts for gender and age [17].

Body composition, reported as body fat percentage, was measured with bioelectric impedance analyses (BIA) using a Tanita body composition analysis scale (Tanita BC-418, Tanita Corporation, Tokyo, Japan).

#### Cardiovascular fitness

The Ekblom-Bak sub-maximal cycle ergometer test was used to estimate cardiovascular fitness (CVF) [18, 19]. To adjust for maturation, the values for prepubertal boys were calculated using the equation for girls [18]. Assessment of puberty was done using the Tanner scale, [20, 21] and boys at stages 1–2 were defined as prepubertal; see Kjellenberg and colleagues [22]. Change in heart rate response to a change in sub-maximal work rate was used to estimate maximal oxygen consumption and expressed as relative values (millilitres of oxygen per kilogramme of body weight per minute, ml/kg/min).

### Outcomes

#### Physical activity and sedentary time

Triaxial accelerometers (Actigraph model GT3X+, LCC, Pensacola, FL, USA) were used to collect information on time spent physically active and sedentary. Participants wore the accelerometer on their right hip for seven consecutive days during all waking hours, except during water-based activities, e.g., showering. At baseline, the first of these 7 days was the day after the participants visited the School of Sport and Health Sciences, while at follow-up either the teacher or the participant indicated the start day. Data were collected at a sampling rate of 30 Hz and were processed in 5-s bouts for analyses using the programme ActiLife version 6.13.3. Non-wear time was classified as 60 or more consecutive minutes of 0 counts, with zero spike tolerance using triaxial data. A filter based on each participant’s reported bedtime and wake time in the questionnaire was used to separate sleep time from wake time. A valid day was classified as having at least 500 minutes of data after removal of non-wear time and application of the filter.

Additionally, a filter based on school schedules was applied to separate schooltime from leisure time during weekdays. Results are reported for 1) weekdays 2) schooltime, 3) leisure time during weekdays and 4) weekends. Each participant needed to have two valid weekdays of measurement at both baseline and follow-up to be included in the results for the first three domains, while one weekend day was required to be included in the results for weekends. The data were categorised into sedentary time (0–100 cpm), LiPA (101–2295 cpm) and MVPA (≥2296 cpm) [23].

Change in PA and sedentary time was calculated by subtracting baseline values from follow-up values. In total. 12 variables were created based on the accelerometer data by using the three intensity levels (sedentary, LiPA, MVPA) across the four time domains (weekdays, school time, leisure time during weekdays, and weekends). All variables are reported as average minutes per day.

#### Sleep

The participants were asked to report their wake and sleep times during weekdays and weekends. The response options were in 30-minute intervals and were used to calculate sleep duration. Change in sleep duration was calculated by subtracting sleep duration at baseline from that at follow-up and is reported in minutes, separately for weekdays and weekends.

#### Screen time

Screen time was determined using the questions “During a normal weekday/weekend day, outside of school, how much time do you spend looking at a screen (not including schoolwork)?” The response alternatives were “no time at all”, “less than 1 hour”, “1–2 hours”, “3–4 hours”, “5–6 hours”, and “7 hours or more”. For ease of comparison with the other outcome variables, the response options were simplified to reflect the midpoint number of minutes i.e. 1–2 hours became 90 minutes. No time at all was counted as 0 minutes, less than 1 hour as 30 minutes and 7 hours or more as 420 minutes. Change in screen time was calculated by subtracting screen time at baseline from that at follow-up and is reported in minutes, separately for weekdays and weekends.

### Statistical analyses

Descriptive data were presented as percentages for categorical variables and means with standard deviations for continuous variables. Differences between participants and drop-outs at baseline were tested using Chi^2^ tests and independent sample t-tests. As the clusters (schools) explained a much of the variation in outcome variables, multilevel linear regression analyses were performed. The models were applied on two levels: the school and the individual, and a random intercept was modelled for each school. Unstandardised betas (β) are reported with 95% confidence intervals (CI). Residuals were plotted and checked for normality. A bootstrapping estimation was performed to produce sensible estimates for standard errors in the multi-level model, as suboptimal fitness of model or heteroscedasticity was detected. Each exposure was explored separately for association with each outcome. The models for anthropometrics and fitness variables were adjusted for gender and parental education. Crude models are reported in supplementary tables 1–2. No imputations for missing data or loss to follow-up were performed. The level of significance was set to *p* < 0.05. The IBM SPSS Statistics for Windows software, version 26 was used for the statistical analyses.

## Results

Just over half of the participants from baseline also participated at follow-up (51.4%), see Table 1. Significantly more girls than boys participated at follow-up (*p* = 0.006) and a higher proportion of participants than drop-outs had both parents born in Sweden (*p* = 0.008).

**Table 1:** Baseline characteristics of participants and drop-outs

	Participants	Drop-outs	*p*-value^1^
	n (%)	n (%)	
Total	585 (51·4)	554 (48·6)	
Gender (Missing *n* = 0)			
Boys	263 (45·0)	295 (53·2)	**0·006**
Girls	321 (55·0)	259 (46·8)	
Parental education (Missing *n* = 37)			
More than 12 years	389 (68·1)	341 (64·2)	0·170
12 years or less	182 (31·9)	190 (35·8)	
Participant country of birth (Missing *n* = 10)			
Sweden	501 (86·1)	466 (85·2)	0·859
Europe	24 (4·1)	22 (4·0)	
Outside Europe	57 (9·8)	59 (10·8)	
Parents’ country of birth (Missing *n* = 32)			
Both parents born in Sweden	357 (62·5)	299 (55·8)	**0·008**
One born outside Sweden	69 (12·1)	99 (18·5)	
Both born outside Sweden	145 (25·4)	138 (25·7)	
BMI status (Missing *n* = 4)			
Underweight/Normal weight	467 (80·2)	437 (79·0)	0·611
Overweight/Obesity	115 (19·8)	116 (21·0)	
MVPA (min/day)			
Weekdays (Missing *n* = 155)	57·9 (20·5)	58·2 (21·1)	0·819
School time (Missing *n* = 155)	26·1 (11·1)	27·0 (11·3)	0·208
Leisure time on weekdays (Missing *n* = 155)	31·8 (14·8)	31·2 (15·2)	0·532
Weekends (Missing *n* = 222)	38·2 (25·6)	37·5 (24·9)	0·678
LiPA (min/day)			
Weekdays (Missing *n* = 155)	148·4 (32·2)	143·5 (33·0)	**0·016**
Schooltime (Missing *n* = 155)	73·0 (18·8)	73·1 (20·6)	0·928
Leisure time on weekdays (Missing *n* = 155)	75·4 (23·5)	70·4 (21·4)	**< 0·001**
Weekends (Missing *n* = 222)	117·6 (37·3)	117·9 (42·3)	0·892
Sedentary (min/day)			
Weekdays (Missing *n* = 155)	618·0 (77·7)	651·4 (80·8)	0·593
Schooltime (Missing *n* = 155)	292·4 (35·8)	291·4 (39·3)	0·682
Leisure time on weekdays (Missing *n* = 155)	325·6 (68·3)	324·0 (70·3)	0·697
Weekends (Missing *n* = 222)	544·8 (80·2)	533·3 (89·3)	**0·040**
Sleep (min/day)			
Weekdays (Missing *n* = 17)	514·2 (54·7)	511·8 (58·4)	0·466
Weekends (Missing *n* = 17)	590·7 (83·6)	597·4 (86·0)	0·188
Screen time (min/day)			
Weekdays (Missing *n* = 13)	197·5 (103·4)	200·3 (99·1)	0·649
Weekends (Missing *n* = 16)	264·3 (109·4)	258·3 (111·1)	0·361
Cardiovascular fitness (CVF, missing *n* = 119)	49·8 (10·2)	49·2 (10·1)	0·386

^1^Chi square test for categorical variables and independent sample t-test for continuous variables

Results in **bold** are significant at α < 0·05

The participants had on average 5.6 (SD = 1.7) valid days of accelerometer wear time at baseline and 3.6 (SD 2.2) valid days at follow-up. A total of 93.1% had at least 3 valid days of wear time at baseline while 63.9% had at least 3 valid days of wear time at follow-up. An increase from a mean of 37 to 41 minutes of MVPA on weekends (*p* = 0.033) was found in the total sample (Table 2). LiPA decreased on weekdays by 23 minutes for girls and 28 minutes for boys (both *p* < 0.001). When weekday school time and leisure time were analysed separately, significant decreases were seen in both time domains for both boys and girls. LiPA on weekends also decreased, by 8 minutes among girls (*p* = 0.008) and 14 minutes among boys (*p* = 0.004).

**Table 2:** Baseline and follow-up results for physical activity and sedentary behaviour variables, stratified by gender

	All participants				Girls				Boys			
		Baseline (2019)	Follow-up (2021)			Baseline (2019)	Follow-up (2021)			Baseline (2019)	Follow-up (2021)	
	n	Mean (SD)	Mean (SD)	*p*-value	n	Mean (SD)	Mean (SD)	*p*-value	n	Mean (SD)	Mean (SD)	*p*-value
MVPA in minutes												
Weekdays	409	58·3 (20·3)	56·1 (25·5)	0·086	248	56·0 (19·6)	54·0 (23·4)	0·228	160	62·2 (20·7)	59·6 (28·2)	0·237
School time	409	26·0 (11·1)	25·4 (12·6)	0·410	248	23·7 (10·4)	23·3 (10·3)	0·588	160	29·5 (11·4)	28·7 (15·0)	0·560
Leisure time on weekdays	409	32·4 (14·7)	30·7 (17·9)	0·076	248	32·2 (14·2)	30·8 (17·0)	0·186	160	32·7 (15·7)	30·8 (19·2)	0·249
Weekends	280	37·1 (23·6)	41·2 (29·9)	**0·033**	175	35·6 (21·9)	40·3 (29·6)	0·055	104	39·8 (26·0)	43·0 (30·3)	0·318
LiPA in minutes												
Weekdays	409	148·3 (31·4)	122·8 (34·3)	**< 0·001**	248	146·1 (31·0)	122·7 (32·9)	**< 0·001**	160	151·8 (31·9)	123·4 (36·3)	**< 0·001**
School time	409	71·6 (18·3)	59·0 (19·7)	**< 0·001**	248	67·1 (16·8)	56·1 (17·7)	**< 0·001**	160	78·8 (18·2)	63·5 (21·7)	**< 0·001**
Leisure time on weekdays	409	76·7 (23·0)	63·9 (24·3)	**< 0·001**	248	79·0 (22·8)	66·6 (23·2)	**< 0·001**	160	73·0 (22·9)	59·9 (25·4)	**< 0·001**
Weekends	280	118·1 (36·9)	107·8 (41·3)	**< 0·001**	175	117·8 (34·5)	109·4 (38·6)	**0·008**	104	119·1 (40·8)	105·6 (45·5)	**0·004**
Sedentary in minutes												
Weekdays	409	623·7 (73·2)	633·1 (84·1)	**0·023**	248	635·2 (68·5)	641·2 (81·5)	0·226	160	605·4 (76·7)	621·0 (87·0)	**0·034**
School time	409	293·1 (34·4)	310·9 (48·4)	**< 0·001**	248	300·5 (34·4)	310·0 (45·9)	**0·001**	160	281·3 (31·1)	311·8 (51·8)	**< 0·001**
Leisure time on weekdays	409	330·6 (63·4)	322·3 (81·0)	**0·032**	248	334·7 (59·2)	331·3 (80·4)	0·449	160	324·1 (69·2)	309·2 (79·8)	**0·030**
Weekends	280	548·0 (79·4)	545·1 (96·2)	0·659	175	544·0 (73·1)	540·0 (95·3)	0·630	104	555·6 (88·7)	553·6 (98·0)	0·853
Sleep in minutes												
Weekdays	557	514·9 (54·2)	487·5 (57·2)	**< 0·001**	310	511·7 (52·2)	479·2 (58·7)	**< 0·001**	246	518·7 (56·6)	498·0 (53·6)	**< 0·001**
Weekends	558	590·6 (82·4)	571·5 (75·9)	**< 0·001**	311	593·9 (75·1)	571·3 (78·7)	**< 0·001**	246	585·2 (88·7)	571·5 (72·5)	**0·024**
Screen time in minutes												
Weekdays	556	197·8 (104·1)	243·3 (101·6)	**< 0·001**	310	199·8 (102·4)	248·5 (96·4)	**< 0·001**	245	195·6 (106·4)	236·4 (107·7)	**< 0·001**
Weekends	553	264·8 (109·9)	307·5 (101·2)	**< 0·001**	310	263·4 (104·3)	311·0 (99·4)	**< 0·001**	242	266·8 (117·2)	303·3 (103·6)	**< 0·001**

Changes from baseline to follow-up tested with paired t-tests

Results in **bold** are significant at α < 0·05

An increase in sedentary time was seen for the whole group on weekdays (*p* = 0.023), as well as during school time (*p* < 0.001) and leisure time (*p* = 0.032). During school time, sedentary time increased 10 minutes among girls (*p* = 0.001) and 31 minutes among boys (*p* < 0.001). However, boys also decreased their sedentary time by 15 minutes (*p* = 0.030) during leisure time while no significant change was found among girls (*p* = 0.449).

Sleep duration significantly decreased in the whole sample by around half an hour on weekdays (*p* < 0.001) and around 20 minutes on weekends (*p* < 0.001), these findings were significant also after stratifying by gender. Screen time significantly increased in the whole sample and among boys and girls separately on both weekdays (*p* < 0.001) and weekends (*p* < 0.001), by approximately 40 minutes.

Compared to boys, girls had 5 minutes (CI:7.6 to 3.1) larger decrease in MVPA, during school time after adjusting for MVPA baseline values (Table 3, see also Supplementary Table 1). Higher cardiovascular fitness at baseline was associated with a greater increase in MVPA, during weekdays (β:0.5 CI:0.2, 0.8), school time (β:0.2 CI:0.0, 0.4) and leisure time on weekdays (β:0.3 CI:0.1, 0.5). Although the whole group increased their MVPA during weekends, those with both parents born outside of Sweden had a smaller increase (β:-8.2 CI:-15.4, − 0.5) compared with those that had both parents born in Sweden.

**Table 3:** Associations between exposures and changes in Moderate-to-Vigorous Physical Activity (MVPA) and Light Physical Activity (LiPA)

	MODERATE-TO-VIGOROUS PHYSICAL ACTIVITY				LIGHT PHYSICAL ACTIVITY			
	Weekdays change (min/day)	School time change (min/day)	Leisure time on weekdays change (min/day)	Weekend change (min/day)	Weekdays change (min/day)	School time change (min/day)	Leisure time on weekdays change (min/day)	Weekend change (min/day)
	β (95% CI)	β (95% CI)	β (95% CI)	β (95% CI)	β (95% CI)	β (95% CI)	β (95% CI)	β (95% CI)
Gender^a^								
Boys	REF	REF	REF	REF	REF	REF	REF	REF
Girls	-4·23 (−9·40, 0·30)	**−5·18 (−7·59, −3·05)**	−0·21 (−3·87, 3·07)	−1·11 (−8·01, 6·56)	− 0·02 (−6·75, 5·29)	**−4·13 (− 7·67, − 0·95)**	3·39 (− 1·05, 7·55)	3·04 (− 8·14, 11·95)
Parental education (SCB)^a^								
More than 12 years	REF	REF	REF	REF	REF	REF	REF	REF
12 years or less	2·43 (−2·56, 7·11)	−0·20 (− 2·58, 2·32)	2·76 (− 0·57, 6·08)	−4·40 (−8·99, 5·05)	4·61 (− 2·41, 12·80)	−0·55 (− 3·66, 3·57)	**5·22 (0·54, 10·39)**	11·07 (− 1·70, 20·65)
Parents’ country of birth ^a^								
Both born in Sweden	REF	REF	REF	REF	REF	REF	REF	REF
One born outside Sweden	0·20 (−6·33, 8·11)	2·08 (−1·33, 5·45)	− 1·96 (− 6·52, 4·24)	−1·18 (− 10·94, 9·71)	2·59 (−7·49, 12·44)	1·99 (−3·09, 7·37)	0·91 (− 6·65, 9·54)	0·83 (− 15·24, 15·20)
Both born outside Sweden	−0·32 (− 6·27, 6·05)	2·17 (− 1·37, 5·79)	− 2·08 (− 6·84, 2·54)	**−8·23 (− 15·43, − 0·53)**	4·20 (− 4·49, 12·26)	1·53 (− 2·94, 6·34)	1·94 (− 4·18, 7·79)	2·92 (−13·91, 11·77)
BMI categories^b^								
Normal weight/Underweight	REF	REF	REF	REF	REF	REF	REF	REF
Overweight/Obesity	−5·62 (−12·23, 2·27)	−2·95 (− 6·17, 0·00)	− 2·84 (− 7·69, 2·81)	2·41 (− 7·26, 16·96)	−2·04 (− 10·38, 7·86)	−2·44 (− 6·90, 1·69)	0·50 (− 5·89, 7·30)	0·98 (− 12·51, 14·42)
BMI sds^b^	−1·74 (− 3·67, 0·35)	−0·90 (− 1·85, 0·08)	− 0·97 (− 2·49, 0·65)	−0·31 (− 3·04, 3·09)	0·05 (− 2·92, 2·88)	0·03 (−1·53, 1·52)	−0·12 (− 1·76, 1·64)	0·80 (− 4·07, 4·24)
Body fat in %^b^	− 0·38 (− 0·80, 0·01)	−0·14 (− 0·33, 0·03)	−0·25 (− 0·55, 0·08)	0·18 (− 0·41, 0·88)	−0·26 (− 0·71, 0·25)	−0·05 (− 0·30, 0·23)	−0·23 (− 0·53, 0·17)	0·07 (− 0·78, 0·82)
Cardiovascular fitness (CVF)^b^	**0·49 (0·19, 0·83)**	**0·18 (0·04, 0·35)**	**0·32 (0·11, 0·54)**	−0·08 (− 0·62, 0·33)	0·01 (− 0·42, 0·37)	−0·03 (− 0·24, 0·18)	0·04 (− 0·23, 0·29)	− 0·52 (− 1·09, 0·14)

^a^Adjusted for baseline values of the outcome

^b^Adjusted for baseline values of the outcome, gender, and parental education

Results in **bold** are significant at α < 0.05

Girls decreased their LiPA school time from baseline to follow-up to a greater extent than boys (β:-4.1 CI: − 7.7, − 1.0), see Table 3. While the whole sample decreased LiPA, those with parents with shorter education had lesser of a decrease by 5 minutes (CI:0.5, 10.4) than those who had parents with longer education.

A greater increase in sedentary school time by approximately 15 more minutes (CI: 4.7, 25.0) was seen for those with overweight or obesity at baseline compared to those with normal weight or underweight (Table 4). Each one-unit increase in BMI sds was associated with a n increase of almost 5 minutes of sedentary school time (CI:1.8, 7.7). An increase of one percentage point in body fat was associated with a greater increase in sedentary school time by 0.8 minutes (CI:0.2, 1.3). An additional baseline cardiovascular fitness of one ml/kg/min was associated with a decrease in weekday sedentary time (β:-1.8 CI:-2.6, − 0.9) as well as sedentary time during leisure time on weekdays (β:-1.4 CI:-2.2, − 0.4).

**Table 4:** Associations between exposures and changes in sedentary time, sleep and screen time

	SEDENTARY TIME				SLEEP DURATION		SCREEN TIME	
	Weekdays change (min/day)	School time change (min/day)	Leisure time on weekdays change (min/day)	Weekend change (min/day)	Weekdays change (min/day)	Weekend change (min/day)	Weekdays change (min/day)	Weekend change (min/day)
	β (95% CI)	β (95% CI)	β (95% CI)	β (95% CI)	β (95% CI)	β (95% CI)	β (95% CI)	β (95% CI)
Gender^a^								
Boys	REF	REF	REF	REF	REF	REF	REF	REF
Girls	−7·28 (− 8·88, 25·02)	−7·12 (− 15·93, 0·30)	15·37 (− 0·57, 31·38)	− 9·66 (− 29·59, 17·84)	**−17.91 (− 26.34, − 9.04)**	− 2.97 (− 17.96, 8.08)	10.12 (− 9.63, 24.28)	8.93 (− 6.96, 23.82)
Parental education (SCB)^a^								
More than 12 years	REF	REF	REF	REF	REF	REF	REF	REF
12 years or less	− 3·78 (− 23·46, 10·46)	−2·24 (− 11·45, 6·10)	−5·88 (− 22·92, 9·67)	12·36 (− 21·13, 40·18)	− 4.63 (− 14.23, 5.54)	−4.86 (− 19.58, 9.14)	**21.33 (1.48, 37.83)**	0.80 (− 19.86, 18.80)
Parents’ country of birth ^a^								
Both born in Sweden	REF	REF	REF	REF	REF	REF	REF	REF
One born outside Sweden	−16·13 (− 45·89, 6·52)	−4·79 (− 17·88, 6·63)	−12·84 (− 39·40, 10·77)	2·97 (− 24·52, 32·13)	− 5.59 (− 18.14, 6.44)	− 0.21 (− 20.21, 17.01)	8.66 (− 15.88, 35.54)	1.15 (− 19.37, 24.57)
Both born outside Sweden	10·02 (− 8·95, 28·74)	7·05 (− 3·26, 17·80)	4·82 (− 12·35, 20·66)	17·18 (− 12·29, 46·17)	2.79 (− 7.52, 15.88)	13.62 (− 2.70, 32.11)	**− 17.00 (− 41.17, − 0.36)**	−1.11 (− 26.35, 15.86)
BMI categories^b^								
Normal weight/Underweight	REF	REF	REF	REF	REF	REF	REF	REF
Overweight/Obesity	11·85 (7·18, 29·47)	**14·52 (4·69, 25·02)**	−2·42 (− 22·59, 15·40)	−21·05 (− 54·59, 10·32)	0.19 (−10.60, 12.62)	−6.39 (− 22.82, 8.77)	−6.03 (− 25.27, 14.89)	−12.18 (− 33.21, 6.26)
BMI sds^b^	4·33 (− 2·65, 9·94)	**4·73 (1·78, 7·71)**	− 1·15 (− 6·59, 4·59)	−1·48 (− 13·75, 8·35)	−1.53 (− 4.95, 2.38)	− 3.32 (− 8.39, 2.27)	3.03 (− 4.49, 9.17)	0.64 (− 6.38, 5.83)
Body fat in %^b^	0·65 (− 0·46, 1·85)	**0·77 (0·21, 1·31)**	−0·13 (− 1·23, 0·97)	− 0·68 (− 2·92, 1·08)	−0.08 (− 0.53, 0.55)	−0.30 (− 1.46, 0.82)	0.40 (−1.19, 1.28)	0.37 (− 0.94, 1.24)
Cardiovascular fitness (CVF)^b^	**−1·76 (− 2·60, − 0·92)**	−0·45 (− 0·97, 0·00)	**−1·35 (− 2·16, − 0·44)**	0·36 (− 1·34, 1·83)	0.26 (− 0.35, 0.75)	−0.07 (− 0.90, 0.69)	**−1.20 (− 2.20, − 0.02)**	**−1.17 (− 2.24, − 0.11)**

^a^Adjusted for baseline values of the outcome

^b^Adjusted for baseline values of the outcome, gender, and parental education

Results in **bold** are significant at α < 0.05

Girls had a greater decrease in sleep on weekdays than boys, by 18 minutes (CI:-26.3, − 9.0, see Table 4). Those with parents with shorter education increased their screen-time with 21 more minutes than those with parents with longer education (CI:1.5, 37.8) and although both parental background groups increased their weekday screen time, those with both parents born outside of Sweden had less of an increase by 17 minutes (CI:-41.2, − 0.4) than those with both parents born in Sweden. Higher cardiovascular fitness was associated with a decrease in screen time on weekdays (β:1.3 CI:2.3, 0.1).

## Discussion

In this sample of Swedish adolescents, we found that, while MVPA remained virtually stable across the week, LiPA generally decreased and sedentary time increased when comparing data from before the COVID-19 pandemic (fall 2019) and approximately 1 year into the pandemic (spring 2021). We also found that sleep duration decreased and screen time increased. Overall, these findings align with previous research conducted during the pandemic, but a circumstance that should be considered when comparing studies is that the type and extent of restrictions have varied between countries and over time [10–12].

PA has been estimated to decrease with age on average by 4.2% per year in children and adolescents older than 5 years [24]. Interestingly, our analysis showed that MVPA increased by 4 minutes during weekends in the whole sample. This change was in the opposite direction from many other studies that have used self-reported data, [10–12] but also recent accelerometer-based studies [25, 26]. For example, one accelerometer-based study found that MVPA decreased by 17 minutes per day when comparing PA before (spring 2019) and during (spring 2020) the pandemic among 64 children aged 7–12 years in the Netherlands [25]. Another recent study from Wales that included about 500 children and adolescents aged 8–18 years during and after lockdown, showed that MVPA increased by 12 minutes per day when they returned to school [26].

Sweden adopted several COVID-19-related safety recommendations, such as keeping social distance and avoiding large gatherings [13]. This led to cancelled organised sport activities, and indoor sports activities have been greatly affected [27]. One potential explanation for our finding that MVPA remained virtually stable may be that adolescents have undertaken PA and exercise programmes in the home or outdoor environment. In one Swedish report, 40% of adolescents stated that they had exercised at home and outdoors more than usual [27].

There are currently few studies from Sweden that have investigated PA during the pandemic. However, our findings align with those from a previous Swedish cross-sectional study where most of the 8300 children and adolescents self-reported similar PA levels [15]. In the same study, 20% reported that their PA had decreased whereas approximately 15% of those older than 13 years reported increased PA. Another study from Sweden showed no differences in longitudinal changes in PA patterns between cohorts measured exclusively before the pandemic and cohorts measured right before and during the pandemic [28].

Moreover, we observed that daily LiPA decreased by about 10–25 minutes across the week and that both sedentary time (about 9 min/d during weekdays) and non-academic screen time (about 40 min/d) increased. These findings are in line with previous research [25, 26], and studies generally show that both academic and non-academic-related screen time have increased across a wide range of devices and media during the pandemic [10].

Moreover, we found that gender, parental education, markers for overweight and obesity, and CVF predicted changes in the outcome variables considered in this study. Generally, girls, participants with overweight or obesity, those with a higher percentage of body and those with lower CVF experienced less favourable changes in PA patterns. For example, among girls, daily school time MVPA and LiPA decreased more, than among boys. Although not entirely consistent across studies, previous research indicates that girls’ PA may have been more affected during the pandemic than boys’ [10]. The larger decrease in LiPA among girls is also consistent with a previous study based on accelerometer data [25].

Markers for overweight and obesity were associated with an increase in sedentary time during school. We also found that CVF, as measured by a sub-maximal cycle ergometer test, predicted changes in PA patterns and screen time. Higher CVF at baseline was associated with more MVPA and less sedentary time and screen time. The fact that higher fitness is related to favourable changes in PA patterns during the pandemic confirms some previous work that has used self-reported data for PA and various fitness measures [10, 11].

We also observed a concurrent decrease in sleep duration by about 20–30 minutes per day across the week. Girls decreased their sleep on weekdays 18 minutes more than boys. Previous studies during the pandemic have reported mixed findings for sleep duration among adolescents [10]. However, studies among children and adolescents suggest reduced sleep quality as a result from the change in routine during the pandemic [29].

This is the largest study to date that has used accelerometer data to investigate COVID-19-pandemic-related changes in PA patterns among Swedish adolescents. The major strengths of this study are the use of accelerometer-based data for PA, measured both right before the pandemic started in the fall of 2019 and during the pandemic in the spring of 2021. This is an important contribution since virtually all previous studies have relied on self-reported data for PA, making them susceptible to bias and error due to over- or underreporting [30, 31]. In terms of limitations, the non-representative sample of adolescents suggests that caution is needed for generalisation. However, the schools were chosen to maximise variation in the type of municipality and the socioeconomic setting. Another limitation is that the dropouts from the follow-up measurement were significantly more likely to be boys, have one parent born outside Sweden and have less sedentary time and LIPA. These could have led to a slight overestimation of the changes in LIPA and sedentary time.

## Conclusion

In this large study of Swedish adolescents from before and during the COVID-19 pandemic we found unfavourable changes in sedentary time, LiPA, screen time and sleep. Female gender, shorter parental education, lower CVF, overweight or obesity, and higher percent body fat at baseline led to adverse changes on the outcomes. These vulnerable groups need to be supported both in the post-pandemic period but also in the event of other epidemics. However, MVPA remained generally unchanged, perhaps due to the mild restrictions that were implemented in Sweden. As studies from countries that employed more extensive lockdowns show a decrease in MVPA, a milder lockdown strategy might be beneficial from the perspective of maintaining MVPA levels.

## Supplementary Information

**Additional file 1: Supplementary Table 1.** Changes in the MVPA and LiPA by gender, parental education, parental country of birth and BMI categories.

**Additional file 2: Supplementary Table 2.** Changes in sedentary time, sleep and screen time by gender, parental education, parental country of birth and BMI categories.

## Data Availability

The datasets generated and/or analyzed during the current study are not publicly available due in order to protect the confidentiality of the participants but are available from Björg Helgadóttir upon on reasonable request. The data are held at The Swedish School of Sport and Health Sciences.

## Abbreviations

BMI: Body Mass Index
COVID-19: Coronavirus Disease 2019
CVF: Cardiovascular fitness
LiPA: Light Physical Activity
MVPA: Moderate-to-Vigorous Physical activity
PA: Physical Activity
