# Supplementary material for: COVID-19 induced changes in physical activity patterns, screen time and sleep among Swedish adolescents - a cohort study
Source: BMC Public Health. 2023 Feb 23;23:380. doi: 10.1186/s12889-023-15282-x (PMC9947443; doi:10.1186/s12889-023-15282-x)
Supplement: Supplementary file 1 — Additional file 1: Supplementary Table 1. Changes in the MVPA and LiPA by gender, parental education, parental country of birth and BMI categories. [file 12889_2023_15282_MOESM1_ESM.docx]

**Supplementary Table 1.** **Changes in the MVPA and LiPA by gender, parental education, parental country of birth and BMI categories.**

|  | **MODERATE-TO-VIGOROUS PHYSICAL ACTIVITY** | | | | **LIGHT PHYSICAL ACTIVITY** | | | |
| --- | --- | --- | --- | --- | --- | --- | --- | --- |
|  | **Weekdays change (min/day)** | **School time change (min/day)** | **Leisure time on weekdays change (min/day)** | **Weekend change (min/day)** | **Weekdays change (min/day)** | **School time change (min/day)** | **Leisure time on weekdays change (min/day)** | **Weekend change (min/day)** |
|  | Δ (95% CI) | Δ (95% CI) | Δ (95% CI) | Δ (95% CI) | Δ (95% CI) | Δ (95% CI) | Δ (95% CI) | Δ (95% CI) |
| Gender |  |  |  |  |  |  |  |  |
| Boys | -2·6 (-7·0, 1·7) | -0·8 (-3·4, 1·8) | -1·9 (-5·0, 1·3) | 3·2 (-3·1, 9·6) | **-28·4 (-34·1, -22·7)** | **-15·3 (-18·8, -11·8)** | **-13·1 (-17·1, -9·1)** | **-13·4 (-22·5, -4·3)** |
| Girls | -1·9 (-5·1, 1·2) | -0·5 (-2·3, 1·3) | -1·4 (-3·6, 0·7) | 4·7 (-0·1, 9·5) | **-23·4 (-27·8, -19·0)** | **-11·0 (-13·5, -8·5)** | **-12·4 (-15·7, -9·2)** | **-8·3 (-14·5, -2·2)** |
|  |  |  |  |  |  |  |  |  |
| Parental education (SCB) |  |  |  |  |  |  |  |  |
| More than 12 years | -2·9 (-6·0, 0·2) | -0·1 (-1·8, 1·6) | **-2·8 (-4·9, -0·6)** | 4·5 (-0·0, 9·0) | **-25·9 (-30·0, -21·9)** | **-11·5 (-13·9, -9·1)** | **-14·5 (-17·3, -11·6)** | **-15·4 (-21·2, -9·6)** |
| 12 years or less | -0·7 (-5·3, 3·9) | -2·0 (-5·0, 1·0) | 1·3 (-1·9, 4·5) | 3·0 (-4·5, 10·5) | **-25·2 (-32·2, -18·2)** | **-15·9 (-20·0, -11·8)** | **-9·3 (-14·4, -4·2)** | 3·0 (-7·9, 13·9) |
|  |  |  |  |  |  |  |  |  |
| Parents’ country of birth |  |  |  |  |  |  |  |  |
| Both born in Sweden | -1·8 (-5·0, 1·4) | -0·7 (-2·6, 1·1) | -1·1 (-3·4, 1·1) | 5·0 (-0·0, 10·0) | **-26·5 (-30·8, -22·2)** | **-12·4 (-15·0, -9·8)** | **-14·1 (-17·2, -11·0)** | **-13·7 (-19·9, -7·4)** |
| One born outside Sweden | -4·3 (-11·3, 2·7) | -0·1 (-3·4, 3·2) | -4·2 (-9·8, 1·4) | 2·5 (-9·5, 14·5) | **-24·3 (-35·6, -13·0)** | **-12·8 (-19·1, -6·5)** | **-11·5 (-19·9, -3·2)** | -9·9 (-27·0, 7·3) |
| Both born outside Sweden | -1·5 (-7·1, 4·2) | -0·2 (-3·7, 3·2) | -1·3 (-5·0, 2·4) | 1·9 (-3·8, 7·7) | **-20·9 (-28·1, -13·6)** | **-13·1 (-17·1, -9·1)** | **-7·7 (-12·8, -2·7)** | -0·9 (-11·9, 10·0) |
|  |  |  |  |  |  |  |  |  |
| BMI categories |  |  |  |  |  |  |  |  |
| Normal weight/Underweight | -1·7 (-4·4, 1·0) | -0·4 (-1·9, 1·2) | -1·3 (-3·2, 0·6) | 3·6 (-0·4, 7·6) | **-25·4 (-29·2, -21·6)** | **-12·0 (-14·2, -9·8)** | **-13·4 (-16·1, -10·6)** | **-10·1 (-15·7, -4·5)** |
| Overweight/Obesity | -4·9 (-12·4, 2·5) | -1·9 (-6·1, 2·4) | -3·1 (-8·0, 1·8) | 7·3 (-4·6, 19·1) | **-25·8 (-34·8, -16·8)** | **-16·1 (-21·4, -10·9)** | **-9·7 (-16·1, -3·2)** | -10·5 (-23·4, 2·3) |
| Results in **bold** are significant at α<0.05 | | |  |  |  |  |  |  |
