# Supplementary material for: COVID-19 induced changes in physical activity patterns, screen time and sleep among Swedish adolescents - a cohort study
Source: BMC Public Health. 2023 Feb 23;23:380. doi: 10.1186/s12889-023-15282-x (PMC9947443; doi:10.1186/s12889-023-15282-x)
Supplement: Supplementary file 2 — Additional file 2: Supplementary Table 2. Changes in sedentary time, sleep and screen time by gender, parental education, parental country of birth and BMI categories. [file 12889_2023_15282_MOESM2_ESM.docx]

**Supplementary Table 2. Changes in sedentary time, sleep and screen time by gender, parental education, parental country of birth and BMI categories.**

|  | **Sedentary time** | | | | **Sleep duration** | | **Screen time** | |
| --- | --- | --- | --- | --- | --- | --- | --- | --- |
|  | **Weekdays change (min/day)** | **School time change (min/day)** | **Leisure time on weekdays change (min/day)** | **Weekend change (min/day)** | **Weekdays change (min/day)** | **Weekend change (min/day)** | **Weekdays change (min/day)** | **Weekend change (min/day)** |
|  | Δ (95% CI) | Δ (95% CI) | Δ (95% CI) | Δ (95% CI) | Δ (95% CI) | Δ (95% CI) | Δ (95% CI) | Δ (95% CI) |
| Gender^1^ |  |  |  |  |  |  |  |  |
| Boys | **15·6 (1·2, 30·1)** | **30·5 (23·0, 38·0)** | **-14·9 (-28·3, -1·4)** | -2·0 (-23·5, 19·5) | **-20·6 (-28·6, -12·6)** | **-13·7 (-25·6, -1·8)** | **40·9 (25·3, 56·5)** | **36·6 (21·6, 51·5)** |
| Girls | 6·0 (-3·8, 15·9) | **9·5 (3·9, 15·0)** | -3·4 (-12·3, 5·5) | -3·9 (-20·0, 12·1) | **-32·5 (-39·0, -26·1)** | **-22·6 (-33·1, -12·2)** | **48·7 (35·8, 61·5)** | **47·6 (34·9, 60·3)** |
|  |  |  |  |  |  |  |  |  |
| Parental education (SCB)^1^ |  |  |  |  |  |  |  |  |
| More than 12 years | **11·2 (1·2, 21·3)** | **17·7 (11·9, 23·4)** | -6·5 (-15·5, 2·6) | -6·9 (-21·7, 8·0) | **-29·7 (-35·6, -23·7)** | **-20·3 (-28·9, -11·8)** | **43·0 (32·1, 53·9)** | **47·3 (36·2, 58·3)** |
| 12 years or less | 7·9 (-7·0, 22·8) | **18·4 (10·6, 26·2)** | -10·5 (-25·1, 4·1) | 10·5 (-16·3, 37·2) | **-25·8 (-35·1, -16·5)** | **-21·8 (-38·8, -4·7)** | **57·5 (36·5, 78·6)** | **34·3 (15·5, 53·0)** |
|  |  |  |  |  |  |  |  |  |
| Parents’ country of birth ^1^ |  |  |  |  |  |  |  |  |
| Both born in Sweden | 9·7 (-0·7, 20·1) | **17·3 (11·7, 22·8)** | -7·5 (-17·0, 1·9) | -5·9 (-21·9, 10·1) | **-27·4 (-33·5, -21·2)** | **-22·8 (-32·1, -13·4)** | **44·5 (32·7, 56·4)** | **42·9 (31·0, 54·8)** |
| One born outside Sweden | -2·5 (-29·7, 24·7) | 15·4 (-0·2, 30·9) | -17·9 (-42·6, 6·9) | -8·6 (-35·7, 18·5) | **-34·2 (-48·5, -19·8)** | -15·9 (-35·5, 3·6) | **59·5 (33·4, 85·6)** | **46·2 (24·4, 67·9)** |
| Both born outside Sweden | 14·6 (-1·9, 31·1) | **17·9 (8·1, 27·6)** | -3·2 (-18·7, 12·2) | 19·4 (-10·2, 49·0) | **-25·5 (-37·1, -13·9)** | -12·9 (-31·5, 5·8) | **46·2 (22·6, 69·7)** | **42·8 (19·9, 65·8)** |
|  |  |  |  |  |  |  |  |  |
| BMI categories^2^ |  |  |  |  |  |  |  |  |
| Normal weight/Underweight | 6·9 (-1·9, 15·8) | **16·2 (11·2, 21·2)** | **-9·3 (-17·4, -1·2)** | 2·9 (-11·0, 16·8) | -27·7 (-33·3, 22·0) | **-19·9 (-28·4, -11·4)** | **49·3 (38·1, 60·4)** | **49·9 (39·0, 60·8)** |
| Overweight/Obesity | **24·6 (2·8, 46·5)** | **26·1 (14·6, 37·5)** | -1·4 (-22·5, 19·6) | **-34·9 (-67·5, -2·3)** | **-27·5 (-39·0, -15·9)** | -16·0 (-36·8, 4·7) | **27·7 (5·9, 49·5)** | 14·3 (-5·8, 34·4) |
|  |  |  |  |  |  |  |  |  |
